# Health System Capacity to Deliver Routine Childhood Immunisations: An Assessment of Health Facilities in Gazelle and Kokopo Districts, East New Britain

**DOI:** 10.3390/vaccines14090755

**Published:** 2026-08-30

**Authors:** Milena Dalton, Stefanie Vaccher, Christopher Morgan, Benjamin Sanderson, Pele Melepia, Hannah A. James, Lynette Duwaba, Benjamin Paivu, Patrick Kiromat, Elsie Stanley, Edward Waramin, Moses Laman, Caroline S. E. Homer, Michelle J. L. Scoullar, Leanne J. Robinson, Margie Danchin, William Pomat

**Affiliations:** 1 Burnet Institute, 85 Commercial Rd, Melbourne, VIC 3004, Australia; 2 Faculty of Medicine, Dentistry and Health Sciences, University of Melbourne, 161 Barry St., Carlton, VIC 3010, Australia; 3 School of Population and Global Health, University of Melbourne, 207 Bouverie St., Carlton, VIC 3053, Australia; 4 Mustard Seed Global, 54 Shearwater Dr, Glenmore Park, Sydney, NSW 2745, Australia; 5 Burnet Institute, Section 90, Lot 1 Takubar, Kokopo P.O. Box 1458, Papua New Guinea; 6 East New Britain Provincial Health Authority, Butuwin, Kokopo P.O. Box 714, Papua New Guinea; 7 Papua New Guinea National Department of Health, Aopi Building Centre, Waigani Dr, National Capital District 121, Port Moresby P.O. Box 807, Papua New Guinea; 8 Papua New Guinea Institute of Medical Research, Boroko, National Capital District 111, Port Moresby P.O. Box 5854, Papua New Guinea; 9 Papua New Guinea Institute of Medical Research, Goroka P.O. Box 60, Papua New Guinea; 10 The School of Public Health and Preventive Medicine, Monash University, 553 St. Kilda Road, Melbourne, VIC 3004, Australia; 11 Department of General Medicine, The Royal Children’s Hospital, 50 Flemington Road, Parkville, VIC 3052, Australia; 12 Vaccine Uptake Group, Murdoch Children’s Research Institute, Royal Children’s Hospital, 50 Flemington Road, Parkville, VIC 3052, Australia; 13 Kirby Institute, Wallace Wurth Building, High St., University of New South Wales, Sydney, NSW 2052, Australia

**Keywords:** routine immunisation, health facility assessment, Papua New Guinea

## Abstract

Background: Over the past decade, the East New Britain Provincial Health Authority in Papua New Guinea has worked with partner research organisations to implement data-driven interventions to improve immunisation coverage. This paper reports on facility assessments conducted as part of a cross-sectional study to understand improvements and identify enablers and barriers to routine childhood immunisation in the province. Methods: Between May and August 2023, nine urban and rural health facilities, providing immunisation services at the time of the study in five areas, were assessed using an adapted World Health Organisation (WHO) Service Availability and Readiness Assessment tool. The tool evaluated facility characteristics, routine childhood immunisation readiness and vaccine supply data. Data were analysed using descriptive statistics and findings were mapped to adapted WHO health system categories to identify service delivery improvement priorities. Findings: Several aspects of immunisation service availability and quality had improved since an assessment in 2016/17. Strengths included fixed-facility and outreach service models tailored to catchment populations, high availability of cold chain and vaccine administration equipment, improved immunisation guidance and job-aids. Priority areas included increasing workforce capacity, improving health record completion to inform outreach, increasing availability of guidelines and reporting tools, reducing stock-outs and enhancing stock tracking. Conclusions: Offering accessible vaccination at every opportunity and using quality data to inform programme implementation and resource allocation are priorities for the Provincial Health Authority. This facility-level assessment identified existing immunisation delivery strengths and highlighted community- and provincial-level priority investment areas to strengthen facility capacity and routine immunisation service delivery.

## 1. Introduction

A strong health system is critical to achieving and maintaining high routine childhood immunisation coverage as an essential component of primary healthcare [1]. Globally, deaths from vaccine-preventable diseases have declined substantially since the World Health Organisation (WHO) established the Expanded Programme on Immunisation in 1974 across all WHO regions [2]. However, vaccination coverage rates continue to vary across regions, and there remains a large number of zero-dose and under-immunised children globally [3]. In 2023, the year this study was conducted, there were 14.5 million zero-dose children worldwide and an additional 6.5 million estimated to be under-immunised [4]. There has been some progress in reducing this number globally, with an estimated 14.3 million zero-dose children and 5.6 million under-immunised in 2024 [5].

The Expanded Programme on Immunisation, now known as the Essential Programme on Immunisation, was introduced in Papua New Guinea (PNG) in 1981 and includes 11 routine vaccines (Table 1) [6,7]. This programme is delivered by health authorities in each of the 21 provinces and by the Department of Health in the Autonomous Region of Bougainville [6,8]. There are six levels in the PNG health system, from level one (aid posts in the wards) to level six (national referral hospital located in the capital, Port Moresby) [9]. The availability of facilities at each level differs significantly across the country and is influenced by geography, catchment population size, resource availability and programmatic needs [10]. In areas outside Port Moresby, immunisation services are delivered across the five levels at facilities managed by government or faith-based organisations [10,11]. These health facilities reach about 40% of the population with facility-based immunisation services [10,11]. For the majority of the population, immunisation outreach services at the community level are the primary point of access for routine immunisation [11]. Overnight outreach patrols are delivered by rural-based facilities only, and one-day mobile outreach clinics from both urban- and rural-based facilities [12,13].

Since 2021, guided by the National Immunisation Strategy [14] and the National Health Plan [9,15], the PNG National Department of Health and Provincial Health Authorities have progressed initiatives to increase routine childhood immunisation coverage. These include health-system-strengthening activities with a focus on human resources, vaccine supply and management, and service delivery to reduce missed opportunities for vaccination and vaccination dropout rates [14].

PNG has some of the lowest childhood vaccination rates in the world, ranking among the 10 lowest globally for first-dose diphtheria, tetanus, pertussis (DTP1) and the lowest in the Western Pacific Region for DTP3 [5,16,17]. Coverage has generally improved since 2019, despite a COVID-19-related dip in 2021 (DTP1: 52%, DTP3: 39%, first-dose measles–rubella (MR1): 38%) [18,19]. By 2024, coverage recovered to 60% (DTP1), 47% (DTP3), and 44% (MR1) [18,19]. A similar pattern appeared in East New Britain Province: DTP3 and MR1 rose sharply from 2019 to 2020, then declined to 45% each by 2023, before recovering to 67% and 59% in 2024 [20]. DTP1 coverage in the province stayed relatively stable, ranging between 60% and 72% over the same period [20].

Improving data quality and use of data to strengthen immunisation coverage in East New Britain Province is a key priority for the Provincial Health Authority. Since 2014, the East New Britain Provincial Health Authority has been working with partner research organisations to identify and implement data-driven service delivery interventions to improve reproductive, maternal, neonatal and child health in the province as part of the Healthy Mothers, Healthy Babies initiative [21]. This includes strengthening areas of the routine immunisation programme that were identified through a cross-sectional assessment of 12 rural health facilities, staff and clients in the province in 2016/17 [22]. The aim of this paper is to report on health facility assessments conducted as part of a cross-sectional study to understand improvements and provide new evidence on the enablers and barriers to routine childhood immunisation in areas of East New Britain Province [7].

## 2. Methods

### 2.1. Study Design and Setting

Between May and August 2023, a health facility assessment was conducted in nine health facilities in two districts, Gazelle and Kokopo, in East New Britain Province as part of a cross-sectional study to understand improvements and identify enablers and barriers to routine childhood immunisation in the province [7]. East New Britain Province, located on New Britain Island, north-east of the main island of PNG, is the most populous province in the Islands region with 457,169 people [23]. Approximately 65% of these people reside in Kokopo and Gazelle districts [23]. Kokopo is a semi-urban district, home to the provincial capital and includes the Duke of York Islands, while Gazelle is a geographically diverse rural district [24].

Using health facility coverage data, population estimates, and consultation with the East New Britain Provincial Health Authority, we mapped zero-dose and under-immunised children across the province. This identified five local-level government areas (one urban, four rural) for inclusion in the study. These areas had varying levels of DTP1, DTP3 and MR1 coverage and included Inland Baining Rural and Toma-Vunadidir Rural local-level government areas in Gazelle District, and Bitapaka Rural, Duke of York Rural, and Kokopo/Vunamami Urban local-level government areas in Kokopo District [7,25]. The design followed the STROBE statement (Supplementary File S1) [26].

### 2.2. Study Sites and Participants

Level-two and level-three healthcare facilities were purposefully selected from the five local-level government areas as they were providing routine immunisation services at the time of data collection and provided a mix of governance arrangements (e.g., government- and faith-based-organisation-managed), urban and rural locations, and ecological settings (e.g., coastal and inland). Level-1 healthcare facilities were not providing immunisation services at the aid post at the time of data collection. Level-two healthcare facilities provide outpatient primary healthcare services, ambulatory care, short inpatient stay and maternity care in rural and remote areas [27]. Level-three healthcare facilities provide primary healthcare services, ambulatory care, inpatient maternity, and newborn care in central provincial urban communities [27]. All health facilities were accessible by road or sea. The mix of governance arrangements and settings was selected to minimise geographic bias.

A letter of support from the East New Britain Provincial Health Authority for the health facility assessment was provided to the Officer-in-Charge (senior health facility management) of each facility prior to commencing the assessment. On the day of assessment, the health facility was notified in the morning. The research team (H.A.J., L.D., B.P.) recruited the most senior healthcare professional available at each facility at the time of assessment and informed written consent was obtained prior to collection of health facility data.

### 2.3. Data Collection

Study investigators developed a health facility assessment questionnaire to collect data on health facility capacity to deliver routine immunisation services. This questionnaire was informed by the WHO Service Availability and Readiness Assessment tool [28] and an adaptation of this tool used in Bangladesh [29], and validated through consultation with the East New Britain Provincial Health Authority senior management staff (Supplementary File S2). The WHO Service Availability and Readiness Assessment tool was developed to evaluate and monitor the availability and readiness of health facilities to provide a range of services [28]. Across two workshops, Papua New Guinean research officers were trained to implement the healthcare facility assessment questionnaire. Data were collected at the health facility in-person by three Papua New Guinean research officers (H.A.J., L.D., B.P.) using REDCap software v13.3.0 installed on electronic tablets and hosted on the research organisation’s institutional server [7]. To reduce assessor bias, this standardised tool was applied at each facility. Three health facility assessments were completed in Kokopo District in May and two in June; two health facility assessments were completed in Gazelle in July and two in August. The healthcare facility assessment questionnaire was available in Tok Pisin and English, and used branching logic and validation rules to support data collection quality.

### 2.4. Data Categories and Analysis

The questionnaire collected general information on the facility (categorical data on facility level and management), human resources (e.g., number of staff involved in vaccination, number of staff who have received immunisation training), types of services provided (e.g., categorical data on vaccination days offered by the clinic, number of outreach and mobile patrols in the past six months), availability of guidelines and tools (e.g., categorical data on Essential Programme on Immunisation guidelines), health record availability (e.g., categorical data on child health record book and birth and immunisation records), amenities (e.g., categorical data on power and water supply and hand-washing stations), equipment and commodities (e.g., categorical data on cold chain and vaccine administration equipment), stock management (e.g., categorical data on stock-outs in the past 12 months and vaccine stock tracking system), and supply (e.g., categorical data on vaccine supply available).

Data were exported into Stata v17.0 (StataCorp, College Station, TX, USA) for analysis and assessed for completeness by M.D. (Milena Dalton) and B.S. Descriptive statistics were used to summarise the data on healthcare facility characteristics, immunisation service availability and vaccine supply. Data were reported as percentages, except for staff involved in vaccination, staff trained in immunisation, facility-based service days per week (reported as median and range), and outreach/mobile sessions (reported as totals). All data were stratified by district. Missing data is listed as not reported. Findings were mapped against six health systems categories (i.e., service delivery, health workforce, health information systems, access to essential medicines, financing, and leadership/governance) to identify priority investment areas [30].

These data also informed the co-development of suggested strategies for specific investment areas within the adapted health system categories developed by Mounier-Jack et al. to continue to improve routine childhood immunisation programme coverage and quality in East New Britain Province. This includes strategies that can be implemented at the community and health-facility level and those that the East New Britain Provincial Health Authority could prioritise. These strategies were co-developed as part of an iterative process of consultative meetings involving the government (PNG National Department of Health and East New Britain Provincial Health Authority), research partners (Burnet Institute, PNG Institute of Medical Research, University of PNG, Murdoch Children’s Research Institute), and community members from the five study areas.

## 3. Results

### 3.1. Healthcare Facility Characteristics

Data were collected from nine health facilities in Kokopo and Gazelle districts. The mix of facility governance, location and levels are shown in Table 2. No facilities declined to participate.

### 3.2. Readiness of Health Facilities to Provide Routine Childhood Immunisation Services

The availability of staff involved in vaccination and who had received official immunisation training varied across the two districts. Health facilities in Kokopo reported almost three times more staff involved in vaccination than Gazelle, with a median of seven staff per facility (Table 3). Most of these staff had not received official immunisation training, with a median of three staff trained per facility in Kokopo and two in Gazelle.

Across the two districts, facilities provided a mix of facility-based, overnight outreach and one-day mobile clinic services. In PNG, there is often only one immunisation clinic offered each week at health facilities. We found that one urban and one rural facility in Kokopo district offered immunisation services five days a week [6]. Overall, facilities in Kokopo reported more facility-based service days per week with a median of three days compared to one day in Gazelle.

In the six months before the assessment, four clinics in Kokopo conducted 80 one-day mobile clinic visits combined, compared to 12 across the three clinics in Gazelle; one facility in each district ran none. Most of the mobile clinics in Kokopo were run by a single urban facility. For overnight outreach patrols, three rural Gazelle facilities conducted 15 sessions when combined, versus four across two rural Kokopo facilities; one rural facility in each district conducted none. Availability of guidelines, tools, and health records varied. All relevant guidelines and tools were available in three (75%) Gazelle facilities and two (40%) Kokopo facilities. Child health record books were available for distribution in two (50%) Gazelle facilities and three (60%) Kokopo facilities. All facilities in both districts had paper-based birth and immunisation records, but only three facilities in Kokopo (60%) also had electronic records; one facility in each district had incomplete paper records. Essential amenities were generally present at facilities. All nine facilities reported continuous power supply; three facilities in Gazelle (75%) and three in Kokopo (60%) also reported a back-up power supply. All nine facilities reported availability of running water and eight reported availability of hand-washing stations.

Equipment and commodities were mostly available. All facilities had functioning temperature-monitoring devices, cold box carriers, ice packs, and sharps containers, and all but one in Kokopo had a functioning vaccine refrigerator. The most frequently out-of-stock items were 10 mL and 0.5 mL syringes (missing in two (40%) Kokopo facilities) and adrenaline (missing in one (25%) Gazelle facility).

### 3.3. Vaccine Supply Management

**Figure 1:**
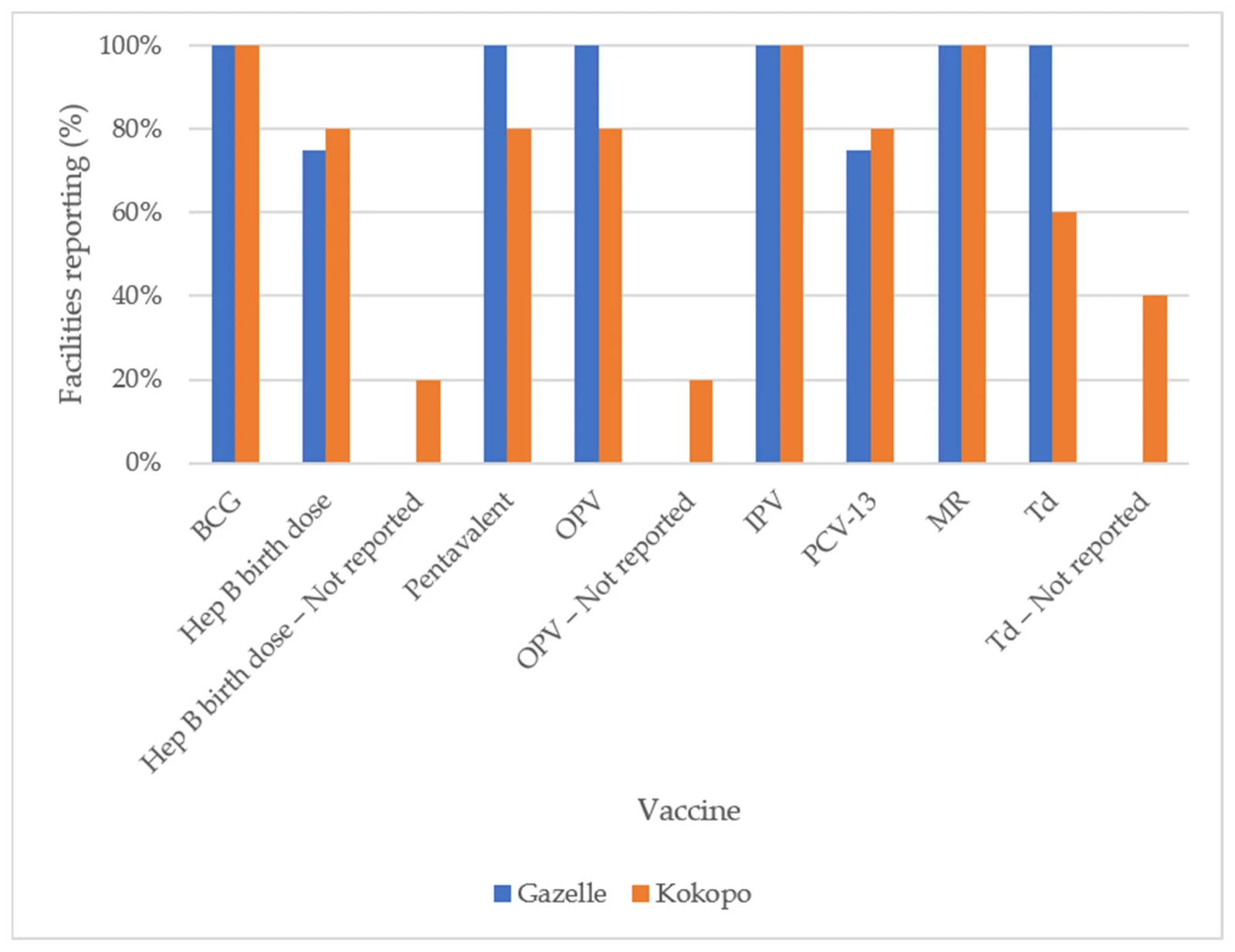
Vaccine stock available on the day of assessment (%). BCG = Bacillus Calmette–Guérin; Hep B = hepatitis B; IPV = inactivated polio vaccine; pentavalent = DTP; Haemophilus influenzae type b, Hep B; PCV = pneumococcal conjugate vaccine; OPV = oral polio vaccine; MR = measles–rubella; Td = tetanus and diphtheria.

Sufficient vaccine stocks are needed for every vaccination opportunity to be realised. All facilities in Kokopo and half in Gazelle experienced a stock-out or expired vaccine doses in the past 12 months. On the day of assessment, four of the nine health facilities (44%) had one or more vaccines out of stock (Figure 1). All nine facilities reported using the paper-based vaccine stock tracking system (Table 4). One facility in Gazelle (25%) and three in Kokopo (60%) also use the mSupply electronic tracking system.

### 3.4. Mapping Findings by Health System Category

Our questionnaire was based on a standardised tool from WHO, and reviewed against the WHO health system categories [30]: service delivery (facility-based, overnight outreach clinic, one-day mobile clinic); health workforce (staff involved in vaccination, staff who received official immunisation training); health information systems (facility birth and immunisation records); access to essential medicines (vaccines, syringes and adrenaline, cold chain, electronic reporting); leadership and governance (Essential Programme on Immunisation guidelines, Adverse Events Following Immunisation guidelines). It is important to acknowledge the interconnections between these categories. For example, inadequate human resources and cold chain management influence service delivery. Our instrument did not assess facility-financing for the immunisation programme or facility-run childhood immunisation community engagement activities, as we restricted our assessment to examine the readiness of local facilities to provide immunisation services, rather than generate demand for services or reflect on central finance policies.

## 4. Discussion

This study describes the results from an assessment of health facility capacity to provide routine childhood immunisations at nine health facilities in Kokopo and Gazelle districts in East New Britain Province in 2023. Across the health system categories, our assessment found numerous strengths and improvements when compared to a previous study conducted in 2016/17 of similarly representative rural health services. This includes the provision of fixed-facility and outreach service models tailored appropriately to catchment populations, and high availability of essential amenities (power supply, running water, equipment, commodities) including cold chain and vaccine administration equipment. Based on our findings and their interpretation in light of other publications from similar settings, we co-developed priorities for investment across adapted health system categories to ensure vaccination at every opportunity (Table 5). Renewed focus is needed on these areas to improve the service availability and quality that is essential in building community trust in immunisation [31].

Our findings demonstrated variable staff availability, with higher levels in the urban centres than rural sites. The need to attract and retain more healthcare professionals is recognised by the PNG National Department of Health as a key priority to improve population health outcomes in PNG [16]. Our findings align with the overall staff shortages seen across PNG, noting that East New Britain Province has 1.82 healthcare professional service delivery staff per 1000 population [45]. This is below the WHO recommendation of 4.45 healthcare professionals per 1000 population for primary care, but higher than the PNG national average of 1.35 [45,46]. The PNG National Department of Health’s 2023 Sector Performance Annual Review highlights larger healthcare budgets, incentives for remote, rurally located health facility positions, and career development opportunities as a focus for investment [16]. Access to, and participation in, immunisation training for more healthcare professionals across all facility levels would create more opportunity for children to be vaccinated as well as provide career development opportunities [47]. This was shown to be effective in Fiji, where a vaccine education and communication training programme for healthcare workers and communities increased community members’ intention to vaccinate from 41% to 83% [40].

Across the two districts, we found a mix of fixed-facility and outreach service models tailored to the catchment population. Optimising outreach service delivery continues to be a priority for East New Britain Province. The previous cross-sectional study conducted in East New Britain Province in 2016/7 also found that outreach was limited, with only two of the nine fixed facilities assessed conducting outreach with overnight stays for remote areas [22]. In 2023, facilities in East New Britain Province conducted 28 outreach clinics per 1000 population, significantly below the national benchmark of 48 [16]. As 60% of vaccinations administered in PNG are through outreach, this indicates that more outreach services are needed [11].

These health facility assessment results demonstrate a considerable improvement in the availability of immunisation guidance and related job-aids. The findings showed 77% of facilities having Essential Programme on Immunisation guidelines available, compared to just 8% of facilities assessed in 2016/7 [22]. Similarly, for guidance and reporting on Adverse Events Following Immunisation, our study found that majority of facilities had guidelines available, although only half had the correct reporting tool. This provides encouragement that the new national Adverse Events Following Immunisation management system developed as part of the 2011–2015 PNG Comprehensive Multi-Year Plan for National Immunisation Programme is seeing at least partial implementation in East New Britain Province [48].

Our results show that East New Britain Province is performing better in terms of identifying births and keeping up-to-date registers, albeit paper-based, than other settings in PNG [6,22,49,50,51]. This is an improvement from 2016/7, where only one-third of clinics were using child health registers in which administered immunisations are recorded [22]. Birth registers and immunisation registers are critical for understanding immunisation coverage, ensuring sufficient vaccine stock and informing microplans, which outline the activities, resources, timing and locations needed to reach every child in the catchment area with routine vaccines [6,52,53]. Along with microplanning training, renewed focus on using electronic registers to improve the quality and efficiency of capturing individual data is needed [52]. In particular, supporting birth registration is important for better understanding of vaccine coverage at a village level, given that only 13% of births are officially recorded in PNG [38,54].

This study found that, overall, the facilities had adequate and functioning cold chain equipment. This is similar to the 2016/7 East New Britain study which found that 10 out of 12 clinics had appropriate, functioning cold chain equipment [22]. Similarly, the availability of adrenaline (to manage severe allergic reactions) in almost all facilities represents a significant improvement from the earlier 2016/17 assessment in East New Britain Province which was not available in any facility previously [22].

Although we did not collect any data on the Provincial Health Authority’s allocation of resources to health facilities for the immunisation programme, we note the critical importance of sufficient and timely disbursement of funds to health facilities [14,16,55]. We also did not collect any data on the health facilities’ provision of community engagement activities to support the demand for routine childhood immunisation, and note the need for investment in community education and social mobilisation as key components of a functional immunisation programme [14].

Study limitations include selecting only sites accessible by road or sea without law-and-order concerns, which may bias findings toward better-equipped facilities. The small sample size (nine facilities) may also limit generalisability. However, our sample included both government- and faith-based-managed facilities that provide immunisation services in the urban, rural and small island communities within the study areas. This is reflective of the diverse contexts across PNG and increases generalisability to other PNG provinces. Strengths of this study include the rigorous selection of local-level government areas, based on mapping of zero-dose and under-immunised children (using DTP1, DTP3, and MR1 coverage and population data) combined with extensive consultation with the East New Britain Provincial Health Authority and healthcare professionals to guide feasibility and site selection. Comparative analysis of one-day mobile clinics and overnight patrols was limited to total activities completed due to the unavailability of disaggregated denominator data. This means we were not able report outreach sessions per 1000 target children. The facility assessment tool proved useful for identifying priority areas for improvement, though future iterations should also assess financing and community engagement processes.

## 5. Conclusions

Our assessment of health facilities in Gazelle and Kokopo districts of East New Britain Province in PNG demonstrates strengths across the health system categories. The identification of priority areas for health-systems-strengthening that can be implemented at both the community and provincial levels provides an opportunity to optimise health facility capacity and increase childhood routine immunisation coverage in East New Britain Province. From a policy perspective, achieving the Immunisation Agenda 2030 targets will require sustained investment in the priority areas identified in this study, particularly vaccine supply, health workforce training, and electronic record systems alongside stronger community engagement and financing mechanisms at the provincial level. Future research could adapt and apply this health facility assessment tool in partnership with other Provincial Health Authorities across PNG to test its utility in different geographic and health system contexts and to evaluate whether addressing the priority areas identified here leads to measurable improvements in routine childhood immunisation coverage over time.

## Figures and Tables

**Table 1:** Vaccination schedule in PNG.

Vaccine	Age
BCG *(vaccine to prevent TB infection)*	birth
Hepatitis B *(vaccine to prevent hepatitis B infection)*	birth
DTP Hib Hep B (*vaccine to prevent diphtheria, tetanus, pertussis, Haemophilus influenzae type B, and hepatitis B infections)* *	1, 2, 3 months
OPV *(weakened poliovirus given by mouth to prevent polio infection)*	1, 2, 3 months
Pneumococcal *(vaccine to prevent Streptococcus pneumoniae infection)*	1, 2, 3 months
IPV *(inactivated poliovirus given by injection to prevent polio infection)*	3, 9 months
MR *(vaccine to prevent measles and rubella infection)*	6, 9, 18 months
Td *(Tetanus toxoid and diphtheria vaccination to prevent tetanus and diphtheria infection)*	7 and 13 years and during pregnancy

* Pentavalent vaccine.

**Table 2:** Healthcare facility characteristics.

		Frequency (%)	
	Category	Gazelle (*N* = 4)	Kokopo (*N* = 5)
* Facility level	Level-two facility Level-three facility	3 (75) 1 (25)	3 (60) 2 (40)
Managing authority	Government Faith-based organisation	1 (25) 3 (75)	3 (60) 2 (40)
Urban/rural	Urban Rural	0 (0) 4 (100)	2 (40) 3 (60)

* One level-three facility was accredited level-four in 2024 after data collection was completed for this study.

**Table 3:** Assessment of routine childhood immunisation service readiness.

Category		Gazelle (*N* = 4)	Kokopo (*N* = 5)
Human resources	Staff involved in vaccination [Median (Range)] Staff who have received official immunisation training [Median (Range)]	3 (1–5) 2 (1–2)	7 (3–8) 3 (0–4)
Services	Facility-based service days per week [Median (Range)] * Total number of overnight outreach services in the past 6 months [*n*] Total number of one-day mobile services in the past 6 months [*n*]	1 (1–2) 15 12	3 (1–4) 4 80
Guidelines and tools	Essential Programme on Immunisation guidelines present [*n* (%)] Adverse Events Following Immunisation guidelines present [*n* (%)] Adverse Events Following Immunisation reporting tool present [*n* (%)]	4 (100) 3 (75) 3 (75)	3 (60) 4 (80) 2 (40)
Health records	Child health book available [*n* (%)] ^ Facility birth and immunisation records completed [*n* (%)] Paper-based Electronic	2 (50) 4 (100) 0 (0)	3 (60) 5 (100) 3 (60)
Amenities	Continuous fridge power supply [*n* (%)] Back-up fridge power supply [*n* (%)] Running water [*n* (%)] Hand-washing stations [*n* (%)]	4 (100) 3 (75) 4 (100) 4 (100)	5 (100) 3 (60) 5 (100) 4 (80)
Equipment and commodities	Functioning vaccine refrigerator [*n* (%)] Functioning continuous temperature-monitoring device [*n* (%)] Cold box/vaccine carriers [*n* (%)] Ice packs [*n* (%)] Sharps container [*n* (%)] Surgical gloves [*n* (%)] Alcohol swabs [*n* (%)] Syringes [*n* (%)] 10 mL 5 mL 2 mL 0.5 mL	4 (100) 4 (100) 4 (100) 4 (100) 4 (100) 4 (100) 3 (75) 4 (100) 4 (100) 3 (75) 4 (100)	4 (80) 5 (100) 5 (100) 5 (100) 5 (100) 4 (80) 5 (100) 3 (60) 5 (100) 4 (80) 3 (60)
	Adrenaline [*n* (%)]	3 (75)	5 (100)

* *n* = 3 for this row as only rural clinics provide overnight outreach services. ^ Facilities could select both paper-based and electronic records.

**Table 4:** Supply and stock management.

	Frequency (%)	
	Gazelle (*N* = 4)	Kokopo (*N* = 5)
Stock-outs or expired vaccine doses in the past 12 months [*n* (%)]	2 (50)	5 (100)
* Vaccine stock tracking system [*n* (%)]		
mSupply	1 (25)	3 (60)
Paper-based	4 (100)	4 (80)
Not reported	0 (0)	1 (20)

* Multiple answers could be reported.

**Table 5:** Suggested investment areas to continue to improve routine immunisation programme coverage and quality in East New Britain Province.

WHO Health System Category	Community- and Health-Facility-Level Opportunities for Improvement	Provincial Health Authority Priority Areas for Investment
Service delivery	Health facilities work with community to help register and track children to inform microplanning [22].	Support health facility transportation needs for outreach [22]. Support microplanning activities and training [32]. Train and deploy lay health workers [22]. Explore opportunities to further integrate immunisation services with other primary healthcare programmes [14].
Health workforce	Bi-monthly supportive supervision at the health-facility level for clinic and outreach services [33,34].	Review staff roles and functions with a view to optimise allocations and workload [22]. Provide mentoring, supportive supervision and training to district- and health-facility-level staff [14]. Explore and evaluate new training formats (i.e., low-dose, high-frequency, and in-service training) [35,36].
Health information	Construction of paper-based or electronic child health registers using the birth register [32].	Regular review meetings to support data analysis at the district and health-facility level [33].
Access to essential medical products, vaccines and technologies	Strengthen the vaccine supply chain through improving finance and stock records linkages [14].	mSupply and stock-management refresher training [37]. Strengthen the integration of vaccine distribution with other programmes and activities [38].
Health financing	Train health facility Officers-in-Charge on how to plan, budget, receive, spend, account for and report funds to strengthen health facility financial management capacity [39].	Advocate for timely release of health function grants [14].
Leadership and governance	Engage in training opportunities to become vaccine champions and drive community uptake of vaccines [40].	Engage healthcare professionals in leadership development programmes, develop a governance environment that spreads decision-making power and encourages multiple forms of accountability [41]. Advocate for immunisation prioritisation at the national level [14].
Community engagement	Strengthen coordination between healthcare professionals and community and religious leaders [32]. Strengthen community education activities and involvement of male parents or caregivers [22].	Revise information, education and communication materials and roll these out across health facilities and in the community [42,43,44].

## Data Availability

The data presented in this study are available upon request from the corresponding authors. Due to ethical restrictions, they are not publicly available.

## Supplementary Materials

The following supporting information can be downloaded at https://www.mdpi.com/article/10.3390/vaccines14090755/s1, Supplementary File S1: STROBE Statement—Checklist of items that should be included in reports of cross-sectional studies; Supplementary File S2: Health facility assessment tool.

## Abbreviations

The following abbreviations are used in this manuscript:

WHO	World Health Organisation
PNG	Papua New Guinea
DTP	Diphtheria, tetanus and pertussis
BCG	Bacillus Calmette–Guérin
Hep B	Hepatitis B
Hib	Haemophilus influenzae type b
OPV	Oral polio vaccine
IPV	Inactivated polio vaccine
PCV	Pneumococcal conjugate vaccine
MR	Measles–rubella vaccine
Td	Tetanus and diphtheria vaccine
