# Supplementary material for: Health System Capacity to Deliver Routine Childhood Immunisations: An Assessment of Health Facilities in Gazelle and Kokopo Districts, East New Britain
Source: Vaccines (Basel). 2026 Aug 30;14(9):755. doi: 10.3390/vaccines14090755 (PMC13611644; doi:10.3390/vaccines14090755)
Supplement: Supplementary file 1 [file vaccines-14-00755-s001.zip › Supplementary Material File S2. Health Facility Assessment Tool.pdf]

Health facility assessment

Health Facility Assessment ID

____

Please select your name:

◯
◯
◯
◯
◯
◯

LLG

◯ Inland Baining
◯ Vunadidir
◯ Bitapaka
◯ Kokopo Urban
◯ Duke of York

District

◯ Gazelle
◯ Kokopo

Ward name (Inland Baining)

◯ ALAKASAM
◯ AWUNGI
◯ BURIT
◯ GAULIM
◯ IVERE
◯ KADAULUNG N0.2
◯ KADAULUNG S/MENT NO.
◯ KAINAGUNAN
◯ KAMANAKAM
◯ KEREBA
◯ KULIT
◯ LAMARAINAM
◯ LAMBERAIN
◯ LIAGA
◯ MALABUNGA
◯ MALASAET
◯ MANAPKI
◯ MANDRESS SETT
◯ RADINGI
◯ RAGAGA
◯ RANGULIT
◯ RAUNSEPNA
◯ RHUNGAGI
◯ VUDAL
◯ VUNAPALADING NO.1
◯ VUNAPALADING NO.2
◯ YAYAMI
◯ Other

Ward name (Toma-Vunadidir)

◯ BAIE
◯ BITAKAPUK NO. 1
◯ BITAKAPUK NO.2
◯ PAPALABA
◯ RABAGI NO.1
◯ RABAGI NO.2
◯ RABATA
◯ RAPITOK NO.1
◯ RAPITOK NO.2
◯ RAPITOK NO.3
◯ RAPITOK NO.4
◯ RATAVUL
◯ TAGITAGI NO.1
◯ TAGITAGI NO.2
◯ TAMANAIRIK NO.1
◯ TAMANAIRIK NO.2
◯ TANAKA
◯ TAULIL NO.1
◯ TAULIL NO.2
◯ VIVIRAN
◯ VIVIRAN NO.2
◯ VUNADIDIR
◯ VUNAKABI
◯ VUNAKAUR
◯ VUNARARERE
◯ WAIRIKI NO. 2
◯ WAIRIKI NO.1
◯ WAIRIKI NO.3
◯ WAIRIKI NO.4
◯ Other

Ward name (Bitapaka)

◯ BALADA
◯ BILUR
◯ BIRAR
◯ GANAI
◯ KAMAKAMAR
◯ KATAKATAI
◯ KORAI
◯ LONDIP
◯ MAKURAPAU
◯ MALAKUNA
◯ MARMAR
◯ MENEBONBON
◯ RAINAU
◯ RALUBANG
◯ RATAVUL
◯ TABUNA
◯ TAUI NO. 1
◯ TAUI NO.2
◯ TOGORO
◯ ULAVEO
◯ VUNABAUR
◯ WATWAT
◯ Other

Ward name (Kokopo Urban)

◯ BITAGALIP
◯ BITAREBAREBE
◯ GUNANBA
◯ GUNANUR
◯ KABAKAUL
◯ KARAVI
◯ KOKOPO TOWN
◯ LIVUAN
◯ MALAKUNA NO.4
◯ NGUNGUNA
◯ PALAVIRUA
◯ PALNAKAUR
◯ RAMALE
◯ TAKUBAR
◯ TINGANAVUDU
◯ ULAGUNAN
◯ ULAULATAVA
◯ VUNABALBAL
◯ VUNAMAMI
◯ VUNAPOPE
◯ Other

Ward name (Duke of York)

◯ BUTLIVUAN
◯ INOLO
◯ KABABIAI
◯ KABILOMO
◯ KARAWARA
◯ KUMAINA
◯ MAKADA
◯ MAREN
◯ MOLOT
◯ MUALIM
◯ NABUAL
◯ PALPAL
◯ URAKUKUR
◯ UTUAN
◯ VIRIAN
◯ WAIRA
◯ Other

Health Facility Name (Inland Baining)

Health Facility Name (Toma-Vunadidir)

Health Facility Name (Bitapaka)

Health Facility Name (Kokopo Urban)

Health Facility Name (Duke of York)

Services

Outreach services offered by the facility in the past six months

____

Mobile clinic services offered by the facility in the past six months

____

Are facility birth and immunisation records completed?

◯ Yes
◯ No

How are facility birth and immunisation records stored?

(select both if relevant)

☐ Paper-copy
☐ Computer

Staff

Number of vaccinators

Note the number of vaccinators (Vaccinators should hold the relevant qualification: Maternal and Child Health Clinic Nurse or Health Extension Officer; not all health staff will be vaccinators).

____

Days that vaccinators conduct vaccination

☐ Monday
☐ Tuesday
☐ Wednesday
☐ Thursday
☐ Friday
☐ Saturday
☐ Sunday

Note the number of staff trained in EPI

____

Note the title or role of staff (do not include names) who have received EPI training and the date they received their training.

Are the EPI guidelines available?

Make sure you sight the guidelines if available

◯ Yes
◯ No

What is the condition of the EPI guidelines?

◯ New
◯ Used - like new
◯ Used - good
◯ Used - fair
◯ Poor

Are the adverse events following immunisation guidelines available?
Make sure you sight the guidelines if available

◯ Yes
◯ No

What is the condition of the adverse events following immunisation guidelines?

◯ New
◯ Used - like new
◯ Used - good
◯ Used - fair
◯ Poor

Is the adverse events following immunisation reporting tool available?
Make sure you sight the guidelines if available

◯ Yes
◯ No

Equipment: For each piece of equipment, note its condition and whether it is functioning.

Is the vaccine refrigerator functioning?

◯ Yes
◯ No

Is the continuous temperature monitoring device functioning?

◯ Yes
◯ No

What is the current temperature of the vaccine refrigerator?

____

Is there a continuous power supply for the refrigerator?

◯ Yes
◯ No

Is there a back-up power supply for the refrigerator?

◯ Yes
◯ No

Is the back-up power supply working?

◯ Yes
◯ No

How long has it not been working for (in months)?

____

Are cold box/vaccine carriers are available?
If none are available, enter '0'

◯ Yes
◯ No

Number available?

____

Are there ice-packs available?

◯ Yes
◯ No

Number of 10ml single-use standard disposable or auto-disposable syringes in stock

If none are available, enter '0'

____

How long have none been available (in months)?

____

Number of 5ml single-use standard disposable or auto-disposable syringes in stock

If none are available, enter '0'

____

How long have none been available (in months)?

____

Number of 2ml single-use standard disposable or auto-disposable syringes in stock

If none are available, enter '0'

____

How long have none been available (in months)?

____

Number of 0.5ml single-use standard disposable or auto-disposable syringes in stock

If none are available, enter '0'

____

How long have none been available (in months)?

____

Is there a sharps container available?

◯ Yes
◯ No

How long have none been available (in months)?

____

Are copies of the child health book for girls available?

◯ Yes
◯ No

How long have none been available (in months)?

____

Are copies of the child health book for boys available?

◯ Yes
◯ No

How long have none been available (in months)?

____

Is there adrenaline available?

◯ Yes
◯ No

How long have none been available (in months)?

____

Are there any hand-washing stations available?

◯ Yes
◯ No

How long have none been available (in months)?

____

Are there any alcohol swabs available?

◯ Yes
◯ No

How long have none been available (in months)?

____

Is there clean, running water available?

◯ Yes
◯ No

What is the source of clean, running water?

(option to select both if relevant)

☐ Rain water
☐ Central supply

Are there any surgical gloves available?

◯ Yes
◯ No

How long have none been available (in months)?

____

Is there any disinfectant available?

◯ Yes
◯ No

How long has none been available (in months)?

____

Vaccine Availability

Is BCG currently available?

◯ Yes
◯ No

Provide an estimate of the number of BCG doses currently available.

____

How long has BCG not been available?

List in months

____

Is Hep B (birth dose) currently available?

◯ Yes
◯ No

Provide an estimate of the number of Hep B (birth dose) doses currently available.

____

How long has Hep B (birth dose) not been available?

List in months

____

Is OPV currently available?

◯ Yes
◯ No

Provide an estimate of the number of OPV doses currently available.

____

How long has OPV not been available?

List in months

____

Is IPV currently available?

◯ Yes
◯ No

Provide an estimate of the number of IPV doses currently available.

____

How long has IPV not been available?

List in months

____

Is Penta-valent (DTP + HiB + HepB) currently available?

◯ Yes
◯ No

Provide an estimate of the number of Penta-valent (DTP + HiB + HepB) doses currently available.

____

How long has Penta-valent (DTP + HiB + HepB) not been available?

List in months

____

Is PCV-13 currently available?

◯ Yes
◯ No

Provide an estimate of the number of PCV-13 doses currently available.

____

How long has PCV-13 not been available?

List in months

____

Is Measles and Rubella currently available?

◯ Yes
◯ No

Provide an estimate of the number of Measles and Rubella doses currently available.

____

How long has Measles and Rubella not been available?

List in months

____

Is tetanus and diphtheria toxoid (Td) vaccine currently available?

◯ Yes
◯ No

Provide an estimate of the number of tetanus and diphtheria toxoid (Td) doses currently available.

____

How long has tetanus and diphtheria toxoid (Td) not been available?

List in months

____

Has there been any stock-outs or expired vaccine doses in the past twelve months?

◯ Yes
◯ No

How many stock-outs have occurred in the past twelve months?

____

Note the duration of each stock-out in months

How is vaccine stock tracked?

☐ Paper records
☐ mSupply
